# Epiphany root canal sealer prepared with resinous solvent is irritating to rat subcutaneous tissues

**DOI:** 10.4317/medoral.17788

**Published:** 2012-02-09

**Authors:** Élcio Daleffe, José E. Vieira-Ozório, Manoel D Sousa-Neto, Danyel E. da-Cruz-Perez

**Affiliations:** 1 DDS, MSc. DDS, MSc. School of Dentistry, University of Ribeirao Preto (UNAERP), Ribeirao Preto, Sao Paulo, Brazil; 2 DDS, PhD. Department of Restorative Dentistry, School of Dentistry of Ribeirao Preto, University of Sao Paulo (USP), Ribeirao Preto, Sao Paulo, Brazil; 3 DDS, PhD. Department of Clinical and Preventive Dentistry, Oral Pathology Unit, Federal University of Pernambuco, Recife, Pernambuco, Brazil

## Abstract

Objective: This study assessed the biocompatibility of the Epiphany endodontic sealer prepared with resinous solvent of Epiphany system (Thinning resin) in rat subcutaneous tissues. Study Design: Polyethylene tubes were filled with the sealer and 4 groups were established: GI, Epiphany prepared with 1 drop of resinous solvent (RS); GII, Epiphany prepared with 1 drop of RS and photoactivated; GIII, Epiphany associated with self-etch primer and prepared with 1 drop of RS; GIV, Epiphany associated with self-etch primer, prepared with 1 drop of RS and photoactivated. The filled tubes were implanted into 4 different regions of the dorsum of 20 adult male rats. Results: After 7, 14 and 21 days, all groups presented a moderate to severe chronic inflammation, necrosis and foreign-body giant cells. At 42 days, although the intensity of chronic inflammatory reaction decreased, the other features still were observed. Conclusion: The Epiphany sealer prepared with the RS was irritating to rat subcutaneous tissues.

** Key words:**Biocompatibility, Epiphany, methacrylate resin sealer, resinous solvent, root canal sealer.

## Introduction

The biocompatibility of root canal sealers is an essential property for ensuring their good performance and successful endodontic treatment (1-3). In vivo and in vitro studies are important to determine the genotoxicity, cytotoxicity, irritant potential and the biological compatibility of these materials (1-7), mainly that recently introduced to the market.

The Resilon-Epiphany canal filling system is composed by Resilon, a synthetic polycaprolactone polymer, based on a polyester-based polymer that contains dimethacrylates, which can bond to methacrylate-based resin sealers, such as Epiphany, a resin-based dual-cure root canal sealer. This system also uses a self-etch primer that contains sulfonic acid terminated functional monomer, HEMA, water, and polymerization initiator. One of the main characteristics of this system is the formation of a resin monoblock that adheres to the den tin of the radicular canal through intratubular tags (8-10). Owing these features, some studies found greater sealing capacity of this system than other materials, being associated with lower index of apical periodontitis (8,11).

This canal filling system is also followed by a resinous solvent (Epiphany Thinning Resin, Pentron Clinical Technologies, LLC, Wallingford, CT, USA), which is an aqueous solution composed by ethoxylated bisphenol-A-dimethacrylate (EBPADMA) resins with photo-initiators, amines, stabilizers and pigments (12). The manufacturer recommends the use of 1 to 2 drops of this solution in order to adjust the sealer viscosity, when necessary. Rached-Junior et al. (13) observed that the resinous solvent increases the bond strength of Epiphany sealer to dentin walls when photo activated.

According to the manufacturer, the Epiphany sealer is biocompatible and non-cytotoxic. In vivo studies confirmed the good biological properties of the sealer (3,14), even when associated with the self-etch primer that accompanies it (5). However, in vitro tests revealed that the photo activated and fresh sealer presents moderate and severe cytotoxicity, respectively (4,15). The self-etch primer also showed cytotoxicity (4). Moreover, Epiphany showed higher cytotoxicity than other conventional sealers (16) and its citotoxicity depends on the concentration and contact time with the culture cells (17).

Regarding to the solvent resinous that also accompanies the system, there is only one study that shows moderate cytotoxicity of the solution when in contact with HeLa cells (4). To the best of our knowledge, there are not previous in vivo studies that evaluated the biocompatibility of the resinous solvent. Thus, the aim of this study was to evaluate the biocompatibility of the Epiphany root canal sealer prepared with resinous solvent (Thinning resin) in rat subcutaneous connective tissues.

## Material and Methods

This study was previously evaluated and approved by the Animal Research Ethics Committee of the University of Ribeirao Preto, Sao Paulo, Brazil and the ethical concepts for use of laboratory animals were observed in all phases of the experiment. The material assessed was the Epiphany root canal sealer (Pentron®-Clinical Technologies, L.L.C. Wallingford CT., USA), which was associated or not with self-etch primer and prepared or not with the resinous solvent (Epiphany Thinning Resin). According to the preparation of the tested sealer, the following 4 experimental groups were established: GI: Epiphany prepared with 1 drop of resinous solvent; GII: Epiphany prepared with 1 drop of resinous solvent and photo activated; GIII: Epiphany associated with self-etch primer and prepared with 1 drop of resinous solvent; GIV: Epiphany associated with self-etch primer, prepared with 1 drop of resinous solvent and photo activated ( Table 1). The lateral wall of the tubes was used as negative control. For each group, it was used 1.5 cm of the sealer obtained from the mixture of the 2 pastes for 1 drop of the resinous solvent, which was released from the flask positioned perpendicular to the glass plate surface.

**Table 1:**
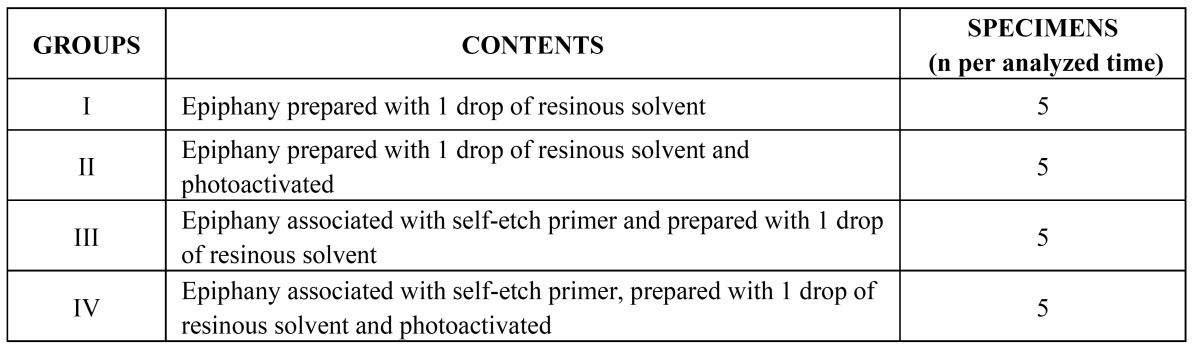
Experimental groups and their respective contents.

For this study, 20 male adult rats (Rattus novergicus, Albinus Wistar), weighing between 200 g and 250 g were used. They were kept in an acclimatized room and received a balanced diet and water ad libitum. The animals were anesthetized by intramuscular injection of ketamine chlorhydrate (0.1 mg/ml) associated with xylazine (0.05 mg/ml), followed by shaving of dorsal fur, disinfection, incision and divulsion of the subcutaneous tissue to insert the test material. To implant the root canal sealer into the rat subcutaneous tissues, sterilized polyethylene tubes 1.2 mm in diameter (0.8 mm internal diameter) and 10 mm long were used. To prevent the sealer from escaping, one of the extremities of the tubes was close by heat. The Epiphany root canal sealer was manipulated in accordance with the manufacturer’s recommendation for clinical use, in a dark room, because it is a dual-cure sealer. In Groups III and IV, a brush was used to coat the internal surface of the polyethylene tubes with the primer supplied with the sealer. The manipulated sealer was carefully put into polyethylene tubes with aid of a paper cone compatible with the diameter of tubes, ensuring that there were no empty spaces and that the sealer did not overflow. Next, the sealer in the tubes was light activated (groups II and IV) and immediately implanted in the subcutaneous tissue in the rat dorsa. The surgical wounds were correctly sutured.

Each animal received four tubes with material, two being in the scapular region and two in the pelvic region (one on the right side and other on the left), with each tube representing a distinct group. After 7, 14, 21 and 42 days, 5 rats were killed by anesthetic overdose and the tubes removed for histological analysis. Thus, there were 5 samples for each experimental group in all the analyzed periods. The specimens were fixed in 10% buffered formalin solution for 24 hours and processed for conventional histological examination.

The connective tissue adjacent to open extremity of each tube was examined in histological cuts (5 µm thick) stained by the hematoxylin and eosin technique, to evaluate the presence or absence of neutrophils, macrophages, lymphocytes, plasma cells, giant foreign body cells, dispersed material and necrotic tissue, classifying each criteria according to quantity in negative (-), slight (+), moderate (++) and intense (+++). Depending on these features, except the presence of dispersed material, the inflammatory reaction of the connective tissue was classified as none to slight (grade I), moderate (grade II), and severe (grade III), according to Campos-Pinto et al. (5).

## Results

In the lateral wall of the tubes (negative control), in all experimental periods, a thin layer of connective tissue without inflammation was observed. The data obtained in the experimental groups are summarized in ( Table 2).

**Table 2:**
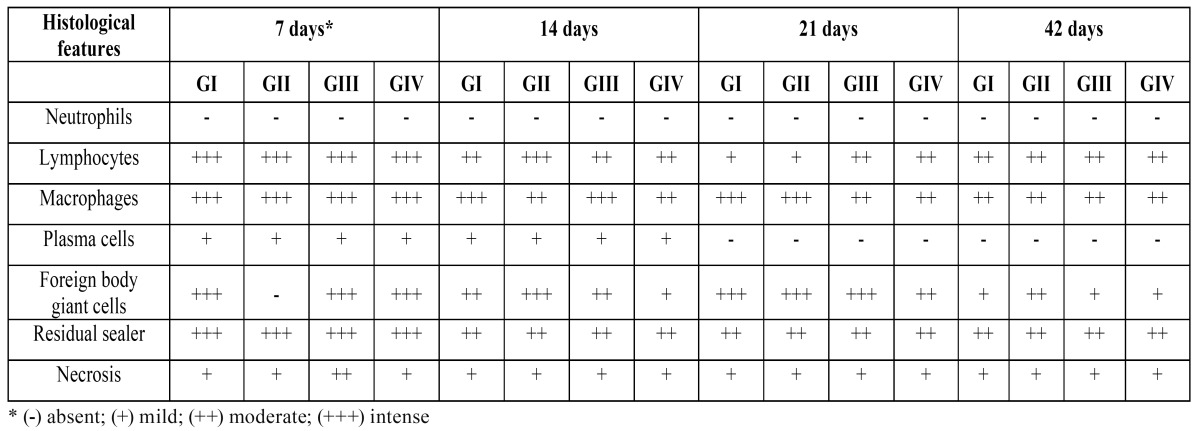
Summary of the histological features in rat subcutaneous tissue after implantation of the Epiphany root canal sealer.

Seven-day results 

**Figure 1:**
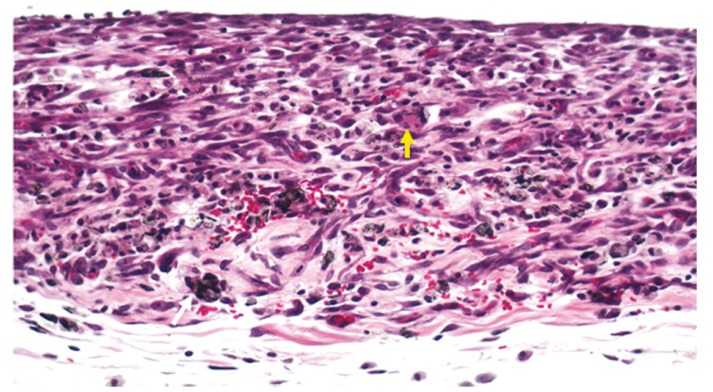
Group III (Epiphany associated with self-etch primer and prepared with 1 drop of resinous solvent), 7 days – Severe chronic inflammatory reaction, residual sealer (white arrow) and foreign body giant cells (yellow arrow) (hematoxylin-eosin, original magnification, x100).

In all groups, a moderate to severe chronic inflammatory reaction was observed, predominantly formed by lymphocytes and macrophages (Fig. 1). There was a large quantity of residual sealer, and small and scarce foci of necrosis. However, in 1 specimen of Group III, an extensive area of necrosis was found. Foreign body giant cells were frequently observed, except in Group II.

14-day results

**Figure 2:**
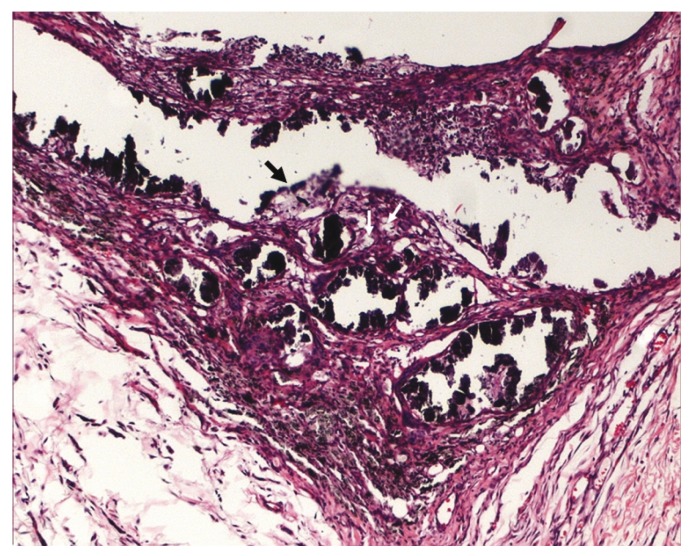
Group IV (Epiphany associated with self-etch primer, prepared with 1 drop of resinous solvent and photoactivated), 14 days – Large quantity of residual sealer beyond the connective tissue adjacent to open extremity of the tube, foci of necrosis (arrows) and severe inflammation (hematoxylin-eosin, original magnification, x100).

A moderate to severe chronic inflammation, formed mainly by lymphocytes and macrophages was observed in all experimental groups, in addition to residual sealer and small foci of necrosis. The residual sealer was observed beyond the connective tissue adjacent to open extremity of the tube, consequently causing more extensive inflammation (Fig. 2). Foreign body giant cells were frequently noted.

21-day results

**Figure 3:**
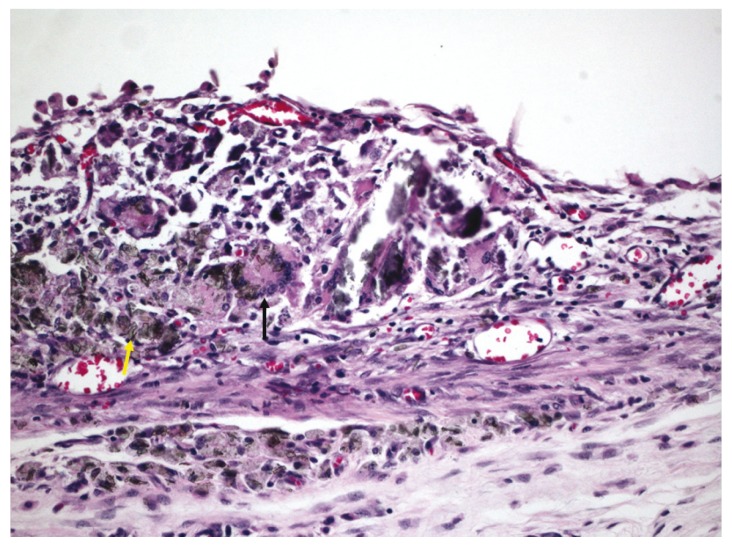
Group I (Epiphany prepared with 1 drop of resinous solvent), 21 days – Large quantity of residual sealer (yellow arrow), with foreign body giant cells (black arrow) (hematoxylin-eosin, original magnification, x100).

After 21 days, all groups presented a moderate to severe chronic inflammation, predominantly formed by lymphocytes and macrophages. Residual sealer, areas of necrosis and foreign body giant cells were also noted (Fig. 3). Moreover, in Group III, there was residual sealer beyond the connective tissue adjacent to open extremity of the tube, and in this area, more extensive inflammation was found.

42-day results

At 42 days, moderate chronic inflammation was observed in all groups, predominantly composed of lymphocytes and macrophages. However, in 1 specimen of Groups I and III, there is a mild inflammatory reaction and in 1 sample of Group IV, a severe inflammation was noted. In all specimens, the connective tissue also presented foci of necrosis, residual sealer and a foreign body granulomatous inflammatory reaction. As had occurred in other groups from previous experimental periods, in Group III there was residual sealer beyond the connective tissue adjacent to open extremity of the tube.

## Discussion

As the manufacturer postulate, some authors also demonstrated that the Epiphany sealer presents low irritability to subcutaneous and bone tissues of animals (3,5,14,18). However, in vitro studies revealed moderate cytotoxicity of this sealer (4,15,17). Moreover, when the cells were exposed to the fresh Epiphany sealer, a greater cytotoxicity was observed (15). With regard to resinous solvent, Merdad et al. (4) found moderate cytotoxicity of this solution when in contact with HeLa cells.

In the present study, after 7, 14 and 21 days, all groups presented connective tissue with moderate to severe chronic inflammatory reaction, necrosis, foreign body giant cells and abundant residual sealer. At 42 days, although there was a slight decrease in the intensity of inflammation (moderate), necrosis, foreign body giant cells and residual sealer still were observed. As the resinous solvent contains similar components to those of the Epiphany sealer, such as ethoxylated bisphenol-A-dimethacrylate (EBPADMA) resins with photo-initiators, amines and stabilizers (12) the sealer prepared with the resinous solvent could eventually increase the concentration of these components and consequently cause irritation and aggression to the tissues. Although Merdad et al. (4) observed a moderate cytotoxicity of the self-etch primer that accompanies the Epiphany system, this substance presented low irritability to subcutaneous tissues of rats when it was used associated with the Epiphany sealer without the resinous solvent (5). Therefore, the tissue irritation observed in the current study is apparently related to the use of the resinous solvent.

Another hypothesis, perhaps the most probable, is based on the fact that the addition of the resinous solvent increased the sealer flow, providing a greater surface of contact with the connective tissue and consequently causing more extensive inflammatory reaction. Rached-Junior et al. (13) observed that the addition of the resinous solvent provided alterations in the Epiphany sealer structure, resulting in monomers of smaller size, which probably increases the sealer flow. In addition, in the present study, residual sealer was observed beyond the connective tissue adjacent to open extremity of the tube in several specimens, suggesting higher flow of the sealer when prepared with the resinous solvent.

A foreign body granulomatous inflammatory reaction associated with abundant residual sealer was frequently observed after all the periods of time analyzed. Considering clinical practice, this is an important finding because, if there were overfilling of Epiphany sealer prepared with the resinous solvent during endodontic therapy, this combination could eventually lead to foreign body granulomatous inflammation in the periapical tissues and favor the persistence of periapical periodontitis (19). However, multi nucleated giant cells was rarely seen when the Epiphany SE sealer (a nonetching version of Epiphany) was used (7).

Although the addition of the resinous solvent to the Epiphany sealer was irritating to subcutaneous tissues in animals, further in vivo and in vitro studies are necessary in order to obtain more information about this canal filling system and to compare the results found in different studies.

## References

[1] Hauman CH, Love RM (2003). Biocompatibility of dental materials used in contemporary endodontic therapy: a review. Part 2. Root-canal-filling materials. Int Endod J.

[2] Bernath M, Szabo J (2003). Tissue reaction initiated by different sealers. Int Endod J.

[3] Sousa CJ, Montes CR, Pascon EA, Loyola AM, Versiani MA (2006). Comparison of the intraosseous biocompatibility of AH Plus, EndoREZ, and Epiphany root canal sealers. J Endod.

[4] Merdad K, Pascon AE, Kulkarni G, Santerre P, Friedman S (2007). Short-term cytotoxicity assessment of the epiphany resin-percha obturating system by indirect and direct contact millipore filter assays. J Endod.

[5] Campos-Pinto MM, de Oliveira DA, Versiani MA, Silva-Sousa YT, de Sousa-Neto MD, da Cruz Perez DE (2008). Assessment of the biocompatibility of Epiphany root canal sealer in rat subcutaneous tissues. Oral Surg Oral Med Oral Pathol Oral Radiol Endod.

[6] Brzovic V, Miletic I, Zeljezic D, Mladinic M, Kasuba V, Ramic S (2009). In vitro genotoxicity of root canal sealers. Int Endod J.

[7] Yamanaka Y, Shigetani Y, Yoshiba K, Yoshiba N, Okiji T (2011). Immunohistochemical analysis of subcutaneous tissue reactions to methacrylate resin-based root canal sealers. Int Endod J.

[8] Shipper G, Orstavik D, Teixeira FB, Trope M (2004). An evaluation of microbial leakage in roots filled with a thermoplastic synthetic polymer-based root canal filling material (Resilon). J Endod.

[9] Ezzie E, Fleury A, Solomon E, Spears R, He J (2006). Efficacy of retreatment techniques for a resin-based root canal obturation material. J Endod.

[10] Versiani MA, Carvalho-Junior JR, Padilha MI, Lacey S, Pascon EA, Sousa-Neto MD (2006). A comparative study of physicochemical properties of AH Plus and Epiphany root canal sealants. Int Endod J.

[11] Shipper G, Teixeira FB, Arnold RR, Trope M (2005). Periapical inflammation after coronal microbial inoculation of dog roots filled with gutta-percha or resilon. J Endod.

[12] Skrtic D, Antonucci JM (2007). Dental composites based on amorphous calcium phosphate - resin composition/physicochemical properties study. J Biomater Appl.

[13] Rached-Junior FJ, Souza-Gabriel AE, Alfredo E, Miranda CE, Silva-Sousa YT, Sousa-Neto MD (2009). Bond strenght of Epiphany sealer prepared with resinous solvent. J Endod.

[14] Onay EO, Ungor M, Ozdemir BH (2007). In vivo evaluation of the biocompatibility of a new resin-based obturation system. Oral Surg Oral Med Oral Pathol Oral Radiol Endod.

[15] Lodienè G, Morisbak E, Bruzell E, Orstavik D (2008). Toxicity evaluation of root canal sealers in vitro. Int Endod J.

[16] Key JE, Rahemtulla FG, Eleazer PD (2006). Cytotoxicity of a new root canal filling material on human gingival fibroblasts. J Endod.

[17] Heitman EP, Joyce AP, McPberson JC, Roberts S, Cbuang A (2008). An in vitro evaluation of the growth of human periodontal ligament fibroblasts after exposure to a methacrylate-based endodontic sealer. J Endod.

[18] Silveira CM, Pinto SC, Zedebski RA, Santos FA, Pilatti GL (2011). Biocompatibility of four root canal sealers: a histopathological evaluation in rat subcutaneous connective tissue. Braz Dent J.

[19] Nair PN (2004 ). Pathogenesis of apical periodontitis and the causes of endodontic failures. Crit Rev Oral Biol Med.

